# Die Impfaktion gegen Poliomyelitis in der DDR im Jahr 1960 am Beispiel der Stadt Halle (Saale): Historische Erfahrungen und Probleme

**DOI:** 10.1007/s00103-022-03538-7

**Published:** 2022-05-16

**Authors:** Anne Thordis Wanke, Florian Bruns

**Affiliations:** 1 Institut für Geschichte und Ethik der Medizin, Profilzentrum Gesundheitswissenschaften, Martin-Luther-Universität Halle-Wittenberg, Halle (Saale), Deutschland; 2 Institut für Geschichte der Medizin, Medizinische Fakultät Carl Gustav Carus, Technische Universität Dresden, Fetscherstraße 74, 01307 Dresden, Deutschland

**Keywords:** Poliomyelitis, Impfung, Sabin-Impfstoff, DDR, Medizingeschichte, Poliomyelitis, Vaccination, Sabin vaccine, German Democratic Republic, History of medicine

## Abstract

In den 1950er-Jahren stellte das epidemische Auftreten der spinalen Kinderlähmung (Poliomyelitis) die Gesundheitssysteme weltweit vor große Herausforderungen. Da eine kausale Therapie der Viruserkrankung nicht existierte, kam der Expositionsprophylaxe eine besondere Bedeutung zu. Letztlich gelang es erst durch die Entwicklung von Impfstoffen, die spinale Kinderlähmung nachhaltig zurückzudrängen. In der Deutschen Demokratischen Republik (DDR) wurde 1960 erstmals in Deutschland die Schluckimpfung nach Sabin-Tschumakow verabreicht, mit der binnen eines Jahres die nahezu vollständige Eradikation der spinalen Kinderlähmung in der DDR gelang. Der Artikel zeichnet anhand von unveröffentlichtem Archivmaterial die systematisch angelegte Impfaktion am Beispiel der damaligen Bezirkshauptstadt Halle (Saale) nach. Allein dort wurden im Mai 1960 innerhalb von 3 Tagen 63.328 Kinder und Jugendliche immunisiert. Bei 78.085 im Vorfeld erfassten Impflingen entsprach dies einer Quote innerhalb der poliovulnerablen Bevölkerungsgruppe von rund 81 %. Die Quellen zeigen, dass die staatliche Struktur des Gesundheitswesens der DDR und das Prinzip der aufsuchenden Impfung zum Erfolg der Impfaktion beitrugen.

## Abstract (en)

In the 1950s, the epidemic occurrence of infantile paralysis (poliomyelitis) posed major challenges to health systems worldwide. Since there was no causal therapy for the viral disease, exposure prophylaxis was of particular importance. Ultimately, it was only through the development of vaccines that infantile paralysis could be permanently reduced. In 1960, the Sabin–Tschumakow oral vaccine was administered in the former German Democratic Republic GDR for the first time in Germany. Within one year, this vaccine succeeded in almost completely eradicating polio in the GDR. The article uses unpublished archival material to trace the systematic vaccination campaign using the example of the then district capital Halle (Saale). There alone, 63,328 children and adolescents were immunized within three days in May 1960. With 78,085 vaccinees recorded in advance, this corresponded to a rate within the polio-vulnerable population group of around 81%. The sources show that the GDR’s government healthcare system and the principle of outreach vaccination contributed to the success of the vaccination campaign.

## Einleitung

Vulnerable Bevölkerungsgruppen möglichst schnell und flächendeckend zu impfen, um die Ausbreitung einer hoch ansteckenden, potenziell tödlichen Viruserkrankung einzudämmen – vor dieser Herausforderung standen Ende der 1950er-Jahre die Gesundheitssysteme im geteilten Deutschland. Was später Geborenen kaum noch erinnerlich ist: Ähnlich wie viele andere Länder weltweit wurden die Bundesrepublik Deutschland (BRD) und die Deutsche Demokratische Republik (DDR) in den 1950er-Jahren wiederkehrend von Poliomyelitisepidemien heimgesucht, die jedes Jahr Hunderte Todesopfer forderten, die meisten davon Kinder und Jugendliche. Allein 1952 verzeichnete man in Westdeutschland 9750 Poliofälle, von denen 778 tödlich endeten [1]. In den USA ließ die Krankheit im selben Jahr über 21.000 Patienten mit bleibenden Lähmungen zurück [2]. Im Sommer 1953 war auch in der DDR ein größerer Ausbruch zu verzeichnen, mehr als 2600 Menschen erkrankten und 141 starben [1, 3]. In den Folgejahren kam es, vorwiegend in den Sommermonaten, sowohl in der DDR als auch in der Bundesrepublik immer wieder zu regionalen Polioepidemien.

Die medizinhistorische Forschung hat sich bisher vor allem den großen Linien der Poliobekämpfung im geteilten Deutschland gewidmet und die jeweils unterschiedlichen gesundheitspolitischen Voraussetzungen und Konzepte herausgearbeitet [4, 5]. Auch der Wissens- und Praxistransfer durch den Eisernen Vorhang hindurch ist untersucht worden [6]. In jüngster Zeit ist zudem eine detaillierte Studie zur speziellen Situation der Poliobekämpfung im geteilten Berlin erschienen [7]. Wie aber sahen die konkreten Schutzmaßnahmen in der DDR aus? Wie wurden sie auf lokaler Ebene organisiert und umgesetzt? Hierüber ist bislang wenig bekannt. Insbesondere der Ablauf der 1960 in der DDR begonnenen ersten Polioschluckimpfungsaktion auf deutschem Boden ist ein Forschungsdesiderat. Im Folgenden gehen wir zunächst kurz auf die Geschichte der Poliomyelitis ein, wobei wir den Schwerpunkt auf die Epidemien der 1950er-Jahre und ihre Bekämpfung in der DDR legen. Dann rekonstruieren wir anhand unveröffentlichter Archivalien und zeitgenössischer Fachliteratur die Impfaktion gegen Poliomyelitis im Jahr 1960 am Beispiel der Stadt Halle (Saale), damals Hauptstadt des gleichnamigen DDR-Bezirks. Im Ergebnis können wir zeigen, dass die Eindämmung epidemischer Infektionskrankheiten auch das zentralistische Gesundheitssystem eines autoritär strukturierten Staates vor große Herausforderungen stellte. Schließlich gelang es jedoch mithilfe einer zügig organisierten und konsequent umgesetzten Impfkampagne, das Auftreten der Poliomyelitis in der DDR in bemerkenswert kurzer Zeit zu beenden.

## Historischer Hintergrund

Im Jahr 1860 prägte der Orthopäde Jakob Heine (1800–1879) die Bezeichnung „spinale Kinderlähmung“ für ein Krankheitsbild, das er einige Jahre zuvor erstmals beschrieben hatte. Der Name verweist bis heute darauf, dass insbesondere Kinder von der Erkrankung betroffen sind und dass es eine seltene, aber gefürchtete paralytische Verlaufsform mit teils irreversiblen schlaffen Lähmungen der Extremitätenmuskulatur gibt. Zunehmendes Wissen über Pathophysiologie und Lokalisation der Krankheit führte 1874 auf Vorschlag von Adolf Kußmaul (1822–1902) zur Bezeichnung Poliomyelitis acuta anterior. 10 Jahre später postulierte Adolf von Strümpell (1853–1925) erstmals den infektiösen Charakter der Poliomyelitis. Dies wurde 1887 durch den schwedischen Arzt Oskar Medin (1847–1927) plausibilisiert und noch vor dem Ersten Weltkrieg durch die Entdeckung des Poliovirus durch Karl Landsteiner (1868–1943) und Erwin Popper (1879–1955) bestätigt [8, 9]. Der fäkal-orale Übertragungsweg stellte sich später als der häufigste heraus. Tödliche Polioverläufe traten meist dann auf, wenn Atemzentrum oder Atemmuskulatur von den Lähmungen betroffen waren.

Durch assistierte Unterdruckbeatmung in der 1929 erstmals eingesetzten „Eisernen Lunge“ konnten seit den 1930er-Jahren auch Patienten mit Atemlähmung vor dem Tod bewahrt werden. Standen nicht genügend dieser röhrenförmigen Geräte zur Verfügung, wie etwa 1952 auf dem Höhepunkt einer sommerlichen Polioepidemie in Dänemark, wurden die Patienten tracheotomiert und mittels Tubus und Beutel beatmet. Hierfür zog das Kopenhagener Blegdam-Hospital seinerzeit Hunderte Medizinstudierende heran, die die Patienten teilweise wochenlang im Schichtbetrieb per Hand beatmeten [10]. In den Folgejahren setzte sich in europäischen Kliniken allmählich die maschinelle Überdruckbeatmung durch, etwa durch Einsatz des 1952 zur Marktreife gebrachten „Poliomaten“ der Firma Dräger [11]. Neben knappen Beatmungskapazitäten beförderten insbesondere die Bilder stark gehbehinderter oder bis zum Hals in den Eisernen Lungen liegender Kinder die gesellschaftliche Angst vor der Poliomyelitis [12]. Ihr unberechenbarer Verlauf, fehlende Therapiemöglichkeiten sowie die möglichen Langzeitfolgen taten ein Übriges, um die Kinderlähmung in den 1950er-Jahren „zur gefürchtetsten Volksseuche der Jetztzeit, zum Schrecken der Mütter“ zu machen ([13], S 7).

Obwohl die Erkrankung statistisch gesehen in der großen Mehrzahl der Fälle leicht oder gar asymptomatisch verlief, beobachteten zeitgenössische Experten die Polioepidemien mit Sorge. Steigende Lebens- und Hygienestandards ließen eine stille Feiung im Kindesalter immer seltener werden, sodass sich Erstinfektionen mit Polioviren in spätere Lebensphasen verschoben. Da sich die Krankheit mit steigendem Alter häufiger klinisch manifestierte, stellte man sich auf stetig steigende Fallzahlen und schwerere Verläufe ein [14]. Daneben wurden eine Pathomorphose der Poliomyelitis bzw. eine erhöhte Pathogenität der auslösenden Viren diskutiert. So glaubten Ärzte eine „Kopfwanderung“ der Krankheit ausmachen zu können, d. h. eine Zunahme spinal- oder bulbärparalytischer Erkrankungsformen mit Lähmung der Schluck- und Atemmuskulatur und entsprechend hoher Letalität [15, 16].

Angesichts der steigenden Krankheitslast wurden international große Anstrengungen zur Bekämpfung der Kinderlähmung unternommen. Bereits der US-amerikanische Präsident Franklin D. Roosevelt (1882–1945), selbst infolge einer Polioerkrankung auf den Rollstuhl angewiesen [17], hatte die Forschung zu dieser Krankheit in seiner Amtszeit stark gefördert. 1938 gründete Roosevelt die National Foundation for Infantile Paralysis, die mittels der „10-Cent-Bewegung“ (March of Dimes) sowohl die Versorgung Erkrankter als auch die Forschung finanziell unterstützte. Nach dem Zweiten Weltkrieg ermöglichten Fortschritte bei der Anzüchtung von Viren die Herstellung größerer Mengen an Polioviren, was die Entwicklung eines Impfstoffs erleichterte. Zudem wiesen Forscher nach, dass 3 unterschiedliche Serotypen des Poliovirus existierten – eine weitere wichtige Erkenntnis für die Impfstoffentwicklung [8].

1955 war schließlich mit der Zulassung des von Jonas Salk (1914–1995) entwickelten Totimpfstoffs in den USA ein bedeutender Meilenstein der Poliomyelitisprophylaxe erreicht. Gegen Ende des Jahrzehnts stand mit dem von Albert Sabin (1906–1993) nahezu gleichzeitig entwickelten Lebendimpfstoff ein weiteres Vakzin zur Verfügung. Der oral verabreichte Lebendimpfstoff vermochte durch die Induktion einer mukosalen Immunität die Virusübertragung effektiver zu unterbrechen als der parenterale Salk-Impfstoff [18]. Weiterentwickelt durch den Virologen Michail Tschumakow (1909–1993) wurde die Sabin-Schluckimpfung seit 1958 millionenfach in der Sowjetunion erprobt. 1961 erfolgte die Zulassung in den USA. Zielgruppe waren in allen Ländern vor allem Kinder und Jugendliche.

Die Bundesrepublik hatte 1957 mit der Immunisierung von Kleinkindern durch den Salk-Impfstoff begonnen. Die Resonanz in der Bevölkerung auf das freiwillige Angebot war jedoch gering und die Impfung daher ohne epidemiologischen Einfluss geblieben. Ein Jahr später folgte die DDR, die den Salk-Impfstoff zunächst aus der Schweiz von der Firma Berna importierte. Die hierfür nötigen finanziellen Mittel mussten durch Einsparungen bei der Vitaminanreicherung von Margarine aufgebracht werden [7]. Ein großer Teil der ersten Impfdosen wurde im Bezirk Halle verimpft ([19], S 25). Später forderten auch andere Bezirke die knappe Vakzine an, um lokale Polioausbrüche einzudämmen, so etwa im Juli 1958 der Bezirk Karl-Marx-Stadt [20].

Ab 1959 standen genügend Chargen aus sowjetischer Produktion zur Verfügung, sodass die Impfkampagne ausgedehnt und den Geburtsjahrgängen 1953 bis 1957 ein Impfangebot gemacht werden konnte. Doch ähnlich wie in der Bundesrepublik zeigte sich ein Großteil der Eltern in der DDR zurückhaltend: Nur 42 % der infrage kommenden Kinder nahmen an der freiwilligen Impfung teil [7]. Während die Salk-Immunisierung in der Bundesrepublik aufgrund von Bedenken verschiedener Behörden und Skepsis in der Bevölkerung auf der Stelle trat [4], schritt das Impfen im ostdeutschen Gesundheitssystem kaum schneller voran. Neben der mangelnden Akzeptanz in der Bevölkerung war dort ein weiterer limitierender Faktor der Mangel an ärztlichem Personal. 1958 hatte die Abwanderung von Ärztinnen und Ärzten in die Bundesrepublik einen Höhepunkt erreicht: 927 Mediziner verließen allein in jenem Jahr die DDR in Richtung Westen [21]. Im Bezirk Halle herrschte Ende der 1950er-Jahre eine besonders geringe Arztdichte. Das entsprechend hohe Arbeitspensum der Mediziner ließ kaum Raum für kurzfristige Impfaktionen [19, 22]. Erst die im Frühjahr 1960 in der DDR eingeführte Schluckimpfung nach Sabin-Tschumakow vermochte der Polioimpfkampagne neuen Schwung zu verleihen, nicht zuletzt, weil ihre Verabreichung weniger ärztliches Personal band. Mit dieser Impfung erreichte die DDR binnen eines Jahres die fast vollständige Eradikation der Poliomyelitis und damit einen deutlichen Vorsprung vor der Bundesrepublik, wo man sich erst 1962 zu einer breiteren Anwendung der Schluckimpfung durchrang [23].

## Poliobekämpfung in der DDR

Angesichts fehlender kausaler Therapiemöglichkeiten spielten vorbeugende Maßnahmen bei der Bekämpfung der Poliomyelitis seit jeher eine zentrale Rolle. Für das stark prophylaktisch orientierte Gesundheitswesen der DDR war die Eindämmung der Poliomyelitis daher ein ideales Handlungsfeld, um seine Stärken unter Beweis zu stellen. Bevor eine Schutzimpfung gegen Polio zur Verfügung stand, konzentrierten sich die Behörden auf klassische seuchenpräventive Maßnahmen wie Aufklärung der Bevölkerung, Isolation der Erkrankten bzw. Quarantäne der Verdachtsfälle, Kontaktnachverfolgung sowie die Vermittlung von Hygieneregeln. Letztere zielten vor allem auf die Körperhygiene und die Desinfektion von Sanitäranlagen ab. Auch lokale Badeverbote wurden verhängt, da Teiche und Seen als typische Übertragungsorte von Polioviren galten.

Die übergeordnete Steuerung dieser und weiterer Seuchenschutzmaßnahmen oblag der 1952 im Ministerium für Gesundheitswesen gebildeten Staatlichen Hygieneinspektion. Aufgabe dieser Einrichtung war es, staatliche Hygieneverordnungen zu erlassen, deren Umsetzung zu überwachen und die regionalen Hygieneinspektionen auf Bezirks‑, Kreis- und kommunaler Ebene anzuleiten. Die vorgesehenen Dienstwege und Abläufe waren allerdings Mitte der 1950er-Jahre wenig eingespielt, da die Bezirke der DDR erst seit Kurzem existierten. 1952 hatte die sozialistische Einheitspartei Deutschlands (SED) die 5 Länderregierungen aufgelöst und durch 15 Bezirke ersetzt, um so einen zentralistischen Einheitsstaat zu schaffen. In der Folgezeit hatte es sich als schwierig erwiesen, die Verwaltungsstellen in den Bezirken und Kreisen mit geeignetem Fachpersonal zu besetzen [24]. Die Polioepidemien der 1950er-Jahre fielen somit in eine Zwischenzeit, in der die Länder bereits aufgelöst, die Strukturen des zentralistischen SED-Staates aber noch nicht voll funktionsfähig waren.

Beraten wurde die Staatliche Hygieneinspektion durch den interdisziplinären Fachausschuss für Poliomyelitis, der 1955 innerhalb des Ministeriums für Gesundheitswesen gegründet worden war [25]. Zum Leiter des Gremiums wurde der Arzt und Poliospezialist Albert Kukowka (1894–1977) bestimmt. Der Internist leitete das Kreiskrankenhaus in Greiz und hatte einige Jahre als Seuchenkommissar für Thüringen gewirkt. Als einer der profiliertesten Polioexperten der DDR entfaltete er eine große publizistische Aktivität. Neben eigener klinischer Erfahrung infolge einer Polioepidemie in Greiz 1951 brachte Kukowka Erkenntnisse mit, die er auf Studienreisen in mehrere europäische Länder gewonnen hatte, darunter Belgien, Frankreich, Großbritannien und die Tschechoslowakei [25, 26]. In seinen zahlreichen Schriften zum Umgang mit Polioepidemien zeigte er eine kritische, aus heutiger Sicht konsequent evidenzbasiert zu nennende Herangehensweise. So stellte er beispielsweise eine lange Negativliste vermeintlicher Heilmittel zusammen, die sich sämtlich als nutzlos im Kampf gegen die Krankheit erwiesen hatten [15]. Auch die Wirkungsmöglichkeiten der Gesundheitsbehörden beurteilte Kukowka auffallend nüchtern. Es sei fraglich, „ob es generell gelingt, einen Vorsprung im Wettlauf mit der Seuche zu erreichen“, zumal Anordnungen und Verbote oftmals nicht eingehalten würden. „Es ist kein Geheimnis, daß die behördlich angeordneten Schutzmaßnahmen in Epidemiegebieten vielfach von der Bevölkerung nicht beachtet werden. Sport‑, Bade- und Tanzverbote in einem bestimmten Gebiet werden dadurch umgangen, daß einzelne, manchmal zahlreiche Bewohner Nachbarorte aufsuchen, in denen keine Verbotstafeln aufgestellt sind“ ([15], S 54). Die Aussicht auf einen effektiven Impfstoff schätzte Kukowka aufgrund der verschiedenen „Spielarten“ des Virus ebenfalls als gering ein ([15], S 51). Glücklicherweise sollte er mit dieser skeptischen Voraussage nicht recht behalten.

Nur wenige Jahre später standen Impfstoffe gegen die Poliomyelitis zur Verfügung. Unter dem Eindruck deutlich steigender Erkrankungszahlen sowie des erstmaligen Einsatzes des Salk-Impfstoffs in der Bundesrepublik im Jahr zuvor hatte 1958 auch die DDR auf Empfehlung des Fachausschusses mit der Beschaffung und Verimpfung der Salk-Vakzine begonnen. Die Wirksamkeit dieses parenteralen Totimpfstoffs galt zwar als begrenzt, doch Vorbehalte gegenüber der Sabin’schen Lebendvakzine ließen die Mediziner in beiden Teilen Deutschlands vorerst am Salk-Impfstoff festhalten. Erst ein Vortrag Tschumakows im Berliner Hygiene-Institut im Februar 1960 änderte dies, zumindest in der DDR. Direkt im Anschluss an den Vortrag gab der von Albert Kukowka geleitete Fachausschuss nach intensiver Diskussion seine Bedenken auf und empfahl die Einführung des Lebendimpfstoffs [25]. Bereits im Folgemonat begann die Staatliche Hygieneinspektion mit der ersten Schluckimpfungsaktion auf deutschem Boden, deren Ablauf am Beispiel des Bezirks Halle nachgezeichnet werden soll.

## Umsetzung der Schluckimpfung in der Bezirkshauptstadt Halle

Nachdem die Schluckimpfungsaktion im März 1960 im Bezirk Dresden mit einer von Tschumakow als Geschenk überlassenen Charge begonnen hatte und gut angenommen worden war [7], erließ das Ministerium für Gesundheitswesen der DDR am 06.04.1960 die „Anordnung zur Bekämpfung der Kinderlähmung“. In dieser wurde festgelegt, dass zwischen April und Juli 1960 flächendeckend die unentgeltliche, freiwillige Immunisierung der „besonders anfälligen Teile der Bevölkerung“ zu erfolgen habe [27]. Hierunter fielen alle Kinder und Jugendlichen im Alter von 2 Monaten bis 20 Jahren. Vorgesehen war die Verabreichung von je 2 Tropfen des Sabin-Tschumakow-Impfstoffs mit etwas Flüssigkeit oder Gebäck. Zunächst sollte gegen Typ I des Virus geimpft werden.

Auf Grundlage der ministeriellen Anordnung begann im Stadtkreis Halle am 06.05.1960 die auf 3 Tage angelegte Impfkampagne. Zur Koordination wurde im Vorfeld ein Operativstab bei der Abteilung Gesundheits- und Sozialwesen des Rates der Stadt gebildet, welcher sich aus Vertretern des staatlichen Gesundheitswesens und der SED-nahen Massenorganisationen zusammensetzte. Unter Vorsitz des Kreisarztes Friedrich Cramer oblag diesem Gremium die Distribution des Impfstoffes an über 78.000 Kinder und Jugendliche [28]. Die Weisung, die Aktion müsse „wegen ihrer politischen Bedeutung und wegen der bereits jetzt angelaufenen Störversuche aus dem Westen als sozialistische Gemeinschaftsarbeit angesehen werden“ und „nach Möglichkeit eine 100 %ige Beteiligung der vorgesehenen Impflinge … erreichen“, offenbart die große politische Bedeutung der Impfkampagne für die DDR [29].

Angesichts der kurzen Vorlaufzeit von 4 Wochen erforderte die Vorbereitung der Immunisierungsaktion eine enge Abstimmung des Operativstabes mit der Universität, den Schulen und Betreuungseinrichtungen sowie den 3 großen Polikliniken der Stadt. Als besonders wichtig wurde die „Popularisierung“ der Impfung angesehen. Plakate an Litfaßsäulen und in Einzelhandelsgeschäften sowie Aufrufe in der lokalen SED-Parteizeitung „Freiheit“ sollten die Bevölkerung von der Teilnahme überzeugen. Die Appelle enthielten keinen Hinweis auf die Freiwilligkeit der Impfung, was in der DDR den Anschein einer staatlichen Anordnung erweckte. Deutlich gewarnt wurde dagegen vor den Risiken einer Infektion: „Nicht selten führt die Erkrankung trotz bester Behandlung zum Tode.“ Die Mütter adressierte man direkt: „… jede Mutti wird so verantwortungsbewußt ihren Kindern gegenüber sein und von ihr [der Impfung] Gebrauch machen“ [30].

Bei der Umsetzung der Impfaktion profitierte der Operativstab von der engen Vernetzung des Gesundheitswesens mit dem Bildungswesen und den großen Industriebetrieben der Stadt. Staatlich angestellte Ärztinnen und Ärzte, Schwestern und Fürsorgerinnen betreuten ohnehin engmaschig die Kinder und Jugendlichen in den städtischen Bildungs- und Betreuungseinrichtungen, was die Durchführung der Impfungen erleichterte. Die Erfassung und Impfung berufstätiger junger Erwachsener gelang relativ leicht durch die zahlreichen betrieblichen Gesundheitseinrichtungen. Neben der Einrichtung von 42 Dauerimpfstellen, an die Impfwillige verwiesen werden konnten, wurden im Stadtkreis Halle überdies 400 mobile „Impftrupps“ gebildet, die Impfungen im Rahmen von Hausbesuchen vornahmen. Ein „Impftrupp“ bestand aus 2 Personen, von denen eine den Impfstoff verabreichte („Tropfer“) und die zweite den Vorgang dokumentierte („Schreiber“) – ein Vorgehen, das sich in den Vorjahren während der sowjetischen Impfkampagne bewährt hatte. Die orale Verabreichung ermöglichte den Verzicht auf ärztliches Personal; für die „Tropfer“ waren lediglich medizinische Vorkenntnisse erwünscht. Entsprechend waren die dafür rekrutierten Freiwilligen zumeist Pflegekräfte, Auszubildende und Angestellte der Gesundheitseinrichtungen sowie Medizinstudierende. Durch mündliche Unterweisungen sowie einen Handzettel mit den wichtigsten Informationen wurden die Impftrupps auf ihren Einsatz vorbereitet. Zusätzlich übten die „Tropfer“ vor Beginn der Aktion das Pipettieren des Impfstoffes mit Wasser [31]. Dass es gelang, ausreichend Freiwillige zu mobilisieren, war für den Halleschen Kreisarzt in mehrfacher Hinsicht ein Erfolg. Zum einen ließ sich damit die gesellschaftliche Akzeptanz der Impfkampagne demonstrieren, zum anderen konnte so auf den Einsatz betrieblicher Gesundheitshelfer verzichtet werden. Letzteres hätte „erhebliche Ausfälle in der Produktion“ nach sich gezogen [28].

Um die Impflinge möglichst lückenlos zu identifizieren, erhielten die Impftrupps die volkspolizeiliche Genehmigung, Einsicht in die Hausbücher nehmen zu dürfen, in denen in der DDR alle Bewohner eines Hauses verzeichnet sein mussten [32]. Insgesamt konnten von den 78.085 im Stadtkreis erfassten Impflingen im Kindes- und Jugendalter innerhalb von 3 Tagen 63.328 erfolgreich geimpft werden, diese Impfrate entsprach 81 % und damit in etwa dem DDR-Durchschnitt. Ein Ergebnis, das „durchaus befriedigen“ könne, wie Kreisarzt Cramer festhielt [28]. Etwa 5000 Impflinge (6,5 %) wurden aus gesundheitlichen Gründen zurückgestellt, 881 (1,1 %) verweigerten die Teilnahme an der Impfung. Rund 11 % konnten an ihrer Wohn- oder Arbeitsstätte nicht angetroffen werden und entgingen so der Impfaktion. Im Abschlussbericht wurde dies mit dem beginnenden Wochenende erklärt, an dem sich zum Beispiel auch viele Studierende nicht in Halle aufhielten. Zusätzlich zu den Kindern und Jugendlichen hatte man als gefährdet eingestufte Erwachsene immunisiert, darunter über 5800 Angestellte des Gesundheits- und Sozialwesens sowie 890 schwangere Frauen.

Über den Stadtkreis Halle hinaus betrachtet verlief die Immunisierungsaktion noch erfolgreicher. In der Woche vom 04.–11.05.1960 erhielten im Bezirk Halle von 590.340 infrage kommenden Kindern und Jugendlichen 536.124 oder 90,8 % die Schluckimpfung, ebenso wie 17.636 gefährdete Erwachsene. Dies lag über der durchschnittlichen Teilnahmequote in der DDR. Im Juni folgte die gemeinsame Impfung gegen die Typen II und III der Poliomyelitiserreger, bei der auch bestehende Impflücken gegen den Typ I weiter geschlossen werden konnten [33].

Probleme traten den Archivquellen zufolge nur vereinzelt auf. So bemängelte Kreisarzt Cramer in seinem Abschlussbericht an das Bezirkshygiene-Institut, dass es in der Stadt trotz entsprechender Anweisung zu wenig Aushänge zur Bekanntmachung der Impfaktion gegeben habe: „Man sah in den Schaufenstern hauptsächlich Plakate für Zirkus, Varieté und Radrennen, aber sehr wenige, die für die Immunisierungsaktion warben“ [28]. Cramer wies ferner darauf hin, dass das im nahe gelegenen Chemiekombinat Bitterfeld hergestellte Wofasept-Seifengelee zur Händedesinfektion nicht ausreichend zur Verfügung stand. Der Deutschen Handelszentrale (DHZ) Pharmazie war es nicht gelungen, die angeforderte Menge kurzfristig bereitzustellen. Der Sabin-Tschumakow-Impfstoff selbst, in Moskau als Konzentrat hergestellt und im Berliner Institut für Immunbiologie verdünnt, war in 8 ml fassende Fläschchen abgefüllt worden. Diese waren einerseits ungleichmäßig gefüllt, was zur Irritation der an der Impfung Beteiligten führte, andererseits reichten sie quantitativ nicht aus, um im Stadtkreis Halle die stationären Impfeinrichtungen und mobilen Impftrupps gleichermaßen zu versorgen [27, 28]. Zugunsten der Durchimpfung der Schulkinder musste daher die Arbeit der Impftrupps am zweiten Tag der Aktion eingeschränkt werden. Impfstoffchargen, die Halle unterstützend aus dem benachbarten Bezirk Leipzig erhalten hatte, trafen am Tag ihres Verfallsdatums ein und enthielten zum Teil bereits getrübte Flüssigkeit. Die lapidare Anweisung, von diesen Chargen dann eben 4 anstatt wie üblich 2 Tropfen zu geben, „trug nicht dazu bei, das Vertrauen zur Immunisierungsaktion gegen Kinderlähmung zu festigen“, kritisierte der Kreisarzt in seinem Abschlussbericht an das Bezirkshygiene-Institut Halle [28]. Weiterhin verwies er auf die „mangelnde Impfbereitschaft“ einiger Personengruppen. So ließen sich von den zur Impfung aufgerufenen Studierenden der Universität Halle-Wittenberg nur knapp 50 % immunisieren.

An die Impfaktion selbst schloss sich eine genaue Überwachung des Gesundheitszustandes der Impflinge an. Als im Stadtkreis Halle bei einigen Schulkindern unspezifische Krankheitssymptome auftraten, wurden diese umgehend untersucht. Hinweise auf einen Zusammenhang der Erkrankungen, die nach Angaben des Kreisarztes „leichterer Art“ waren, ergaben sich nicht [28]. Schwerwiegendere Verdachtsfälle wären dem Bezirkshygienearzt und damit der Staatlichen Hygieneinspektion zu melden gewesen. Da bekannt war, dass die Lebendvakzine bei manchen Impflingen eine Polioerkrankung auszulösen vermochte, wurde dieser Aspekt besonders überwacht. Im Nachgang der Hallenser Impfkampagne vom Mai 1960 wurden weder ernste Nebenwirkungen noch Impfpoliomyelitiden gemeldet. Ab 1961 galt die Weisung des Ministeriums für Gesundheitswesen, bei einem innerhalb von 30 Tagen nach Impfung auftretenden Verdacht auf Impfpolio sowohl bei den Impflingen als auch bei deren Kontaktpersonen Blut- und Stuhlproben in festgelegten Abständen zu entnehmen und diese durch das Institut für Immunbiologie in Berlin auswerten zu lassen [34]. Folgt man einer 1981 publizierten Auswertung von Sieghart Dittmann (*1934), so wurden im Zeitraum 1960–1975 in der DDR 63 atypische Impfverläufe anerkannt, von denen 16 die Kriterien einer Impfpoliomyelitis gemäß Weltgesundheitsorganisation (WHO) erfüllten [35]. Über deren Schwere geben die Quellen keine Auskunft.

Im Ganzen war die Impfkampagne ein großer Erfolg, der in bemerkenswert kurzer Zeit erzielt wurde. Bereits die Poliosaison des Jahres 1960 verlief deutlich glimpflicher als in den Jahren zuvor. Hatte Theodor Kima, Mitglied des Fachausschusses gegen Poliomyelitis und Leiter des staatlichen Institutes für Immunbiologie in Berlin, für den Zeitraum von 1955–1959 durchschnittlich jährlich 1083 Krankheits- und 86 Todesfälle in der DDR errechnet, so traten 1960 nur noch 132 Infektionen mit 8 Todesfällen auf [7]. 1961 wurden 4 Poliomyelitisfälle gemeldet, ein Jahr darauf 3 Infektionen. Seitdem galt die Kinderlähmung in der DDR als „liquidiert“ [36]. Am 13.01.1961 erließ das Ministerium für Gesundheitswesen der DDR eine weitere „Anordnung zur Verhütung der Kinderlähmung“. Darin wurde die Schluckimpfung gegen Kinderlähmung offiziell als Pflichtimpfung für Kinder und Jugendliche und freiwillige Impfung für Erwachsene festgelegt. Einen Zwang zur Impfung gab es jedoch nicht [5]. Allerdings wurde staatlicherseits darauf Wert gelegt, dass alle potenziellen Impflinge den Impftermin zur ärztlichen Vorstellung und Beratung wahrnahmen [37]. Wer nicht erschien, erhielt eine Vorladung zum Kreisarzt. Fälle, in denen Impfungen unter Zwang vorgenommen wurden, lassen sich den Akten nicht entnehmen. Die Durchführung der Impfung mittels koordinierter Immunisierungsaktionen wie der hier vorgestellten im Bezirk Halle wurde in den Folgejahren beibehalten [34].

## Fazit

Impfungen boten Ende der 1950er-Jahre erstmals die Möglichkeit, die weltweite Ausbreitung der Poliomyelitis effektiv zu kontrollieren. Die Bundesrepublik und die DDR verfolgten seinerzeit unter den Bedingungen des Kalten Krieges und angesichts ihrer unterschiedlichen politischen Systeme verschiedene Impfregime. Eine anfängliche Skepsis der Bevölkerung gegenüber neuen Impfstoffen war vor 60 Jahren in beiden Teilen Deutschlands zu verzeichnen. Ungeachtet dessen erreichte das hier am Beispiel des Bezirks Halle betrachtete Gesundheitswesen der DDR durch die systematische Immunisierung der poliovulnerablen Bevölkerungsgruppe mit dem Sabin-Tschumakow-Impfstoff einen durchschlagenden Erfolg in der Poliobekämpfung. Unsere aktengestützte Rekonstruktion der Impfkampagne auf regionaler Ebene zeigt, dass ein Bündel unterschiedlicher Maßnahmen zu einer vergleichsweise hohen Durchimpfungsrate beigetragen hat. Dazu gehörten über Aushänge und Lokalzeitungen vermittelte Appelle an die Bevölkerung, wobei Mütter als spezifische Zielgruppe angesprochen wurden. Weiterhin kam das in der Sowjetunion erprobte Prinzip der aufsuchenden Impfung zur Anwendung, indem aus freiwilligen und medizinisch vorgebildeten Helfern zusammengesetzte Impftrupps die Menschen zu Hause aufsuchten. Vorteilhaft war außerdem, dass die Sabin-Tschumakow-Vakzine als Schluckimpfung vergleichsweise einfach und ohne ärztliche Aufsicht verabreicht werden konnte, was angesichts des in jener Zeit für die DDR und insbesondere für den Bezirk Halle charakteristischen Ärztemangels wichtig war. Erleichtert wurde die in Halle (Saale) innerhalb von 3 Tagen durchgeführte Impfung von über 63.000 Menschen überdies durch Spezifika des staatlichen Gesundheitswesens sowie des politischen Systems der DDR. So waren Schüler und Werktätige über das gut ausgebaute Schul- und Betriebsgesundheitswesen niedrigschwellig zu erreichen. Die in den 15 DDR-Bezirken gleichzeitig und koordiniert stattfindende Immunisierungsaktion profitierte ebenso von der zentralistischen Struktur des staatlichen Gesundheitswesens wie die nachträgliche Überwachung der geimpften Personen. Die aufsuchenden Impftrupps konnten über die sogenannten Hausbücher die in einem Wohngebäude lebenden Personen identifizieren, womit ein Überwachungsinstrument genutzt wurde, das charakteristisch für den erleichterten Zugriff der DDR-Behörden auf den einzelnen Bürger war. Dass aufsuchende Impfungen bei ausgewählten Bevölkerungsgruppen auch in einem demokratischen Rechtsstaat umsetzbar sind, hat die jüngste Gegenwart mit den mobilen COVID-19-Impfteams bewiesen [38]. Bedenklich mutet es nach heutigen Transparenzmaßstäben an, dass die DDR-Bürger im Rahmen der Impfaktion des Jahres 1960 nicht explizit darüber informiert wurden, dass es sich zu diesem Zeitpunkt um eine freiwillige Impfung handelte. Unsere historische Untersuchung zeigt aber auch, dass selbst in einem autoritären Staat wie der DDR eine systematisch angelegte Impfkampagne an Grenzen stieß. Impfstoff war nicht immer in ausreichender Zahl und Qualität verfügbar, nicht alle Bevölkerungsgruppen ließen sich gleichermaßen motivieren und eine lokale Impfquote von mehr als 90 % war eher die Ausnahme als die Regel. Weitere Forschungen sind notwendig, um die hier beschriebene Polioimpfaktion mit anderen Impfkampagnen in der DDR oder darüber hinaus vergleichen zu können.
